# The Small Metal-Binding Protein SmbP Simplifies the Recombinant Expression and Purification of the Antimicrobial Peptide LL-37

**DOI:** 10.3390/antibiotics10101271

**Published:** 2021-10-19

**Authors:** David A. Perez-Perez, Teresa de J. Villanueva-Ramirez, Adriana E. Hernandez-Pedraza, Nestor G. Casillas-Vega, Patricia Gonzalez-Barranco, Xristo Zarate

**Affiliations:** 1 Universidad Autonoma de Nuevo Leon, Facultad de Ciencias Quimicas, Av. Universidad s/n, Cd. Universitaria, San Nicolas de los Garza 66455, Mexico; davidantonio.perez@live.com.mx (D.A.P.-P.); teresa.villanuevarm@uanl.edu.mx (T.d.J.V.-R.); patricia.gonzalezbrn@uanl.edu.mx (P.G.-B.); 2 CHRISTUS—LATAM HUB Center of Excellence and Innovation, S.C., Lazaro Cardenas 2321, San Pedro Garza Garcia 66260, Mexico; adriana.hernandez@christuscei.mx; 3 Universidad Autonoma de Nuevo Leon, Departamento de Patologia Clinica, Hospital Universitario Dr. Jose Eleuterio Gonzalez, Monterrey 64460, Mexico; ncasillasv@uanl.edu.mx

**Keywords:** SmbP, small metal-binding protein, LL-37, recombinant peptides, antimicrobial peptides, *Escherichia coli*, *Staphylococcus aureus*

## Abstract

(1) Background: The cathelicidin peptide LL-37 is a prominent molecule with many biological activities, including antimicrobial. Due to its importance, here, we describe the production of LL-37 tagged with SmbP, a relatively new carrier protein that improves the production of recombinant proteins and peptides in *Escherichia coli*. We present an alternative method for the rapid expression, purification, and antimicrobial evaluation of LL-37, that involves only one purification step. (2) Methods: A DNA construct of SmbP_LL-37 was transformed into *E. coli* BL21(DE3); after overnight expression, the protein was purified directly from the cell lysate using immobilized metal-affinity chromatography. SmbP_LL-37 was treated with Enterokinase to obtain the free LL-37 peptide. The antimicrobial activity of both SmbP_LL-37 and free LL-37 was determined using the colony forming unit assay method. (3) Results: SmbP_LL-37 was observed in the soluble fraction of the cell lysate; after purification with IMAC, protein gel electrophoresis, and analysis by ImageJ, it showed 90% purity. A total of 3.6 mg of SmbP_LL-37 was produced from one liter of cell culture. SmbP_LL-37 and free LL-37 both showed inhibition activity against *Staphylococcus aureus* and *Escherichia coli*. (4) Conclusions: The SmbP fusion protein is a valuable tool for producing biologically-active LL-37 peptide. The production method described here should be of interest for the expression and purification of additional cationic peptides, since it cuts the purification time considerably prior to determination of antimicrobial activity.

## 1. Introduction

The small metal-binding protein SmbP isolated from the periplasm of the bacterium *Nitrosomonas europaea* is an attractive fusion protein for production of recombinant proteins and peptides in *Escherichia coli.* It is a 9.9-kDa protein whose biological function is the expulsion of toxic metal ions from the cell [1]. SmbP improves the expression of recombinant proteins and peptides by increasing their solubility and through avoiding the formation of inclusion bodies. Via its metal-binding capacity, recombinant proteins can be purified through immobilized metal-affinity chromatography (IMAC), obtaining high purities in a single step [2,3]. After purification, proteins are typically digested with the protease Enterokinase to remove SmbP, obtaining the pure target protein or peptide after a second IMAC purification. Due to its low molecular weight, the final yields are enhanced compared to those obtained when other larger fusion proteins are used.

We have previously reported the production of biologically active proteins, such as the human growth hormone, and the antimicrobial peptides (AMPs) Bin1b and VpDef tagged with SmbP, with satisfactory yields and purities [4,5,6]. Interestingly, in the case of the peptide Bin1b, we observed that the presence of SmbP, i.e., the complete SmbP_Bin1b protein construct, did not reduce the ability of the peptide to inhibit the growth of *Staphylococcus aureus* and *Escherichia coli* in its entirety. This feature makes SmbP an attractive carrier protein for the expression of antimicrobial peptides with the same action mechanism as Bin1b (cationic peptides that form pores in the bacterial membrane) [6]. After expression and purification, the recombinant peptide’s antimicrobial activity can be determined without cutting and removing SmbP. Once the activity is confirmed, the free peptide can be obtained after Enterokinase digestion; consequently, the free peptide’s activity is expected to increase, and if desired, a second purification protocol can be applied to obtain the pure peptide, usually another round of IMAC.

The antimicrobial peptide LL-37 is of great interest due to its multiple biological activities, including antimicrobial [7,8], antibiofilm [9], antifungal [10], antiviral [11], and immunomodulatory properties [12,13]. LL-37 forms the C-terminus of the cathelicidin precursor protein hCAP-18. Since this family of proteins is expressed in cells that are in direct contact with the environment, the cathelicidin peptide acts as a primary antimicrobial barrier [14]. Currently, researchers are designing new peptides based on LL-37 to increase its biological activity, such as SAAP-148 [15].

LL-37 has previously been expressed in *Escherichia coli*, using different fusion proteins such as thioredoxin (THX), glutathione S-transferase (GST), and the carbohydrate-binding module III (CBM3) [16,17,18,19]. The previous results show that LL-37 production is challenging, especially its purification, since this requires multiple steps to achieve adequate purity. Therefore, here, we have simplified the complete expression and purification process for the LL-37 peptide using SmbP as the fusion partner, obtaining a recombinant free peptide that preserves antimicrobial activity.

## 2. Results and Discussion

**Figure 1:**
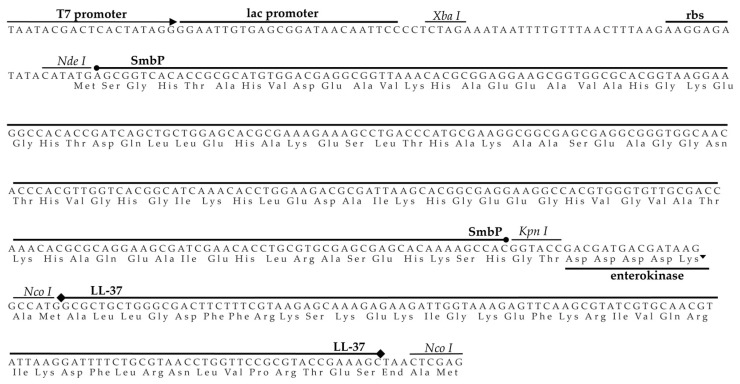
DNA and amino acid sequences for the construct pET30a_SmbP_LL-37.

*Escherichia coli* is one of the most exploited microorganisms for expression of recombinant AMPs, because it can produce significant quantities of recombinant peptides at low cost [20]. However, using *E. coli* as a host can be challenging due to the small size of AMPs, their toxicity to the host cell, and their susceptibility to degradation due to a low molecular weight and high cationic properties [21,22]. For better production in *E. coli*, expression with fusion proteins is a common strategy. Nonetheless, the yields are usually low after tag removal due to their higher molecular weights than the ones from the peptides. Therefore, SmbP, having a 9.9-kDa size, should improve the final yield. Figure 1 shows the DNA and amino acid sequences for the pET30a_SmbP_LL-37 construct. LL-37 was expressed with the fusion protein SmbP at its N-terminus. An Enterokinase recognition sequence was placed between them to allow excision of the free peptide after digestion.

**Figure 2:**
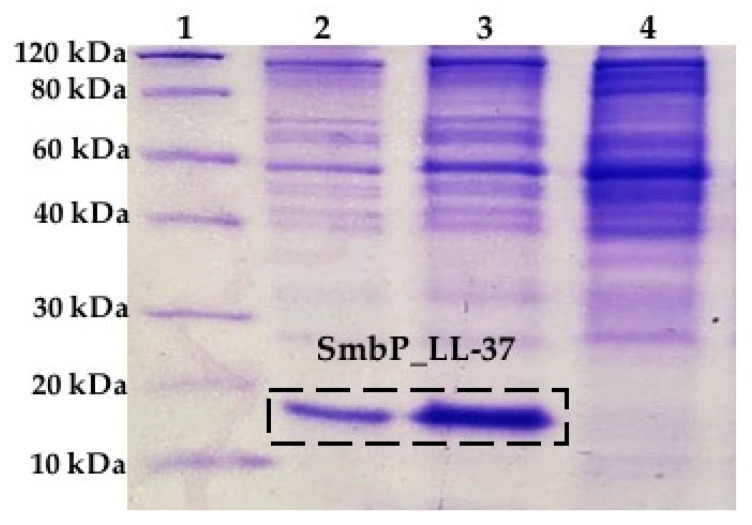
SDS-PAGE (12%) analysis of SmbP_LL-37 small-scale expression. Lane 1: protein marker, Lanes 2 and 3: SmbP_LL-37, and Lane 4: Lysate from *E. coli* BL21(DE3) uninduced cells.

A small-scale expression was performed to evaluate LL-37 production using SmbP as a fusion partner. Figure 2 shows the SDS-PAGE analysis of the cell lysate supernatant for SmbP_LL-37. A new band was observed with a molecular mass of around 15 kDa, indicating that LL-37 tagged with SmbP was expressed in a soluble manner in *E. coli*. We anticipated this result, since a previous work done in our laboratory showed that target recombinant proteins or peptides are primarily found in the soluble fraction with minor or no formation of inclusion bodies [2,6,23].

**Figure 3:**
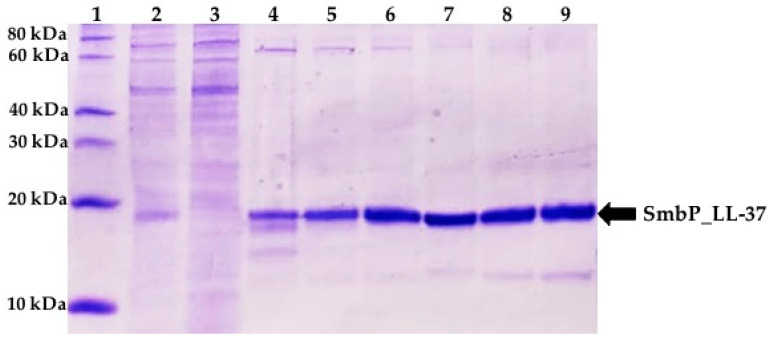
Tricine SDS-PAGE (13.5%) analysis of SmbP_LL-37 after IMAC purification. Lane 1: protein marker, Lane 2: lysate, Lane 3: flowthrough, and Lanes 4–9: elution fractions.

A larger scale expression and purification procedure was then done. Figure 3 shows SDS-PAGE analysis of the elution fractions from the IMAC purification using an imidazole gradient. A total of 3.6 mg of SmbP_LL-37 per liter of culture was obtained with a purity around 90% (as determined by ImageJ) with just one chromatographic step. The purity and amount of protein observed for SmbP_LL-37 were as expected based on the expression and purification of the SmbP-tagged Bin1B and VpDef peptides [5,6]. In the case of LL-37, although the amount of protein was relatively less, the purity was considerably higher.

**Figure 4:**
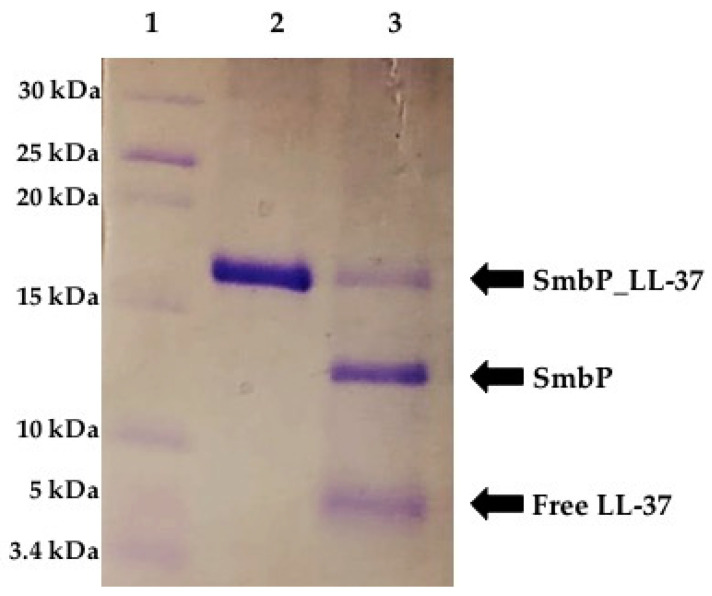
Tricine SDS-PAGE (15%) analysis of SmbP_LL-37 after cleavage with Enterokinase. Lane 1: protein marker, Lane 2: SmbP_LL-37 purified using IMAC, and Lane 3: SmbP_LL-37 after cleavage with Enterokinase.

**Figure 5:**
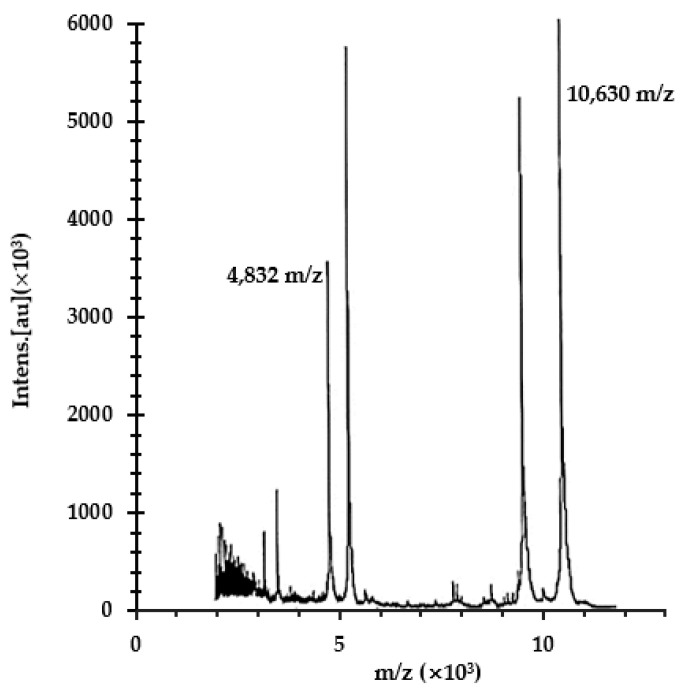
MALDI-TOF MS spectrum of the LL-37 antimicrobial peptide. SmbP_LL-37 was treated with Enterokinase; afterward, a sample was analyzed with the Microflex LT/SH mass spectrometer (Bruker) using α-Cyano-4-hydroxycinnamic acid as the matrix.

Routinely, fusion proteins are cleaved and removed, because they interfere with the biological activity of the recombinant peptides. As mentioned above, from our previous work, SmbP fused to Bin1b did not completely abrogate its antimicrobial activity. Consequently, the complete SmbP_LL-37 protein was used for the antimicrobial assay. However, since we also wanted to test the activity of the free LL-37 peptide, SmbP_LL-37 was digested with Enterokinase overnight. Figure 4 shows the electrophoresis analysis after digestion. Consistent with previous reports, the cut was incomplete, most likely due to LL-37 aggregation, making the cleavage site less accessible [24]. Nevertheless, most of the protein was cut, releasing LL-37, which appeared in the gel at the appropriate size, around 5 kDa. The peptide was further characterized using mass spectrometry with a molecular weight recorded at 4832 Da (Figure 5), in agreement with the calculated value. The MS MALDI-TOF spectrum also showed the molecular weight for SmbP to be 10,630 Da.

We tested the free LL-37 antimicrobial activity without further purification, as a mixture with SmbP, the minimal Enterokinase used, and the remnants of SmbP_LL-37, expecting a higher activity from a free peptide no longer fused to SmbP. Previous results have demonstrated that SmbP has no antimicrobial activity by itself [6]. As for the Enterokinase, control experiments showed that it also lacks antimicrobial activity (data not shown).

**Figure 6:**
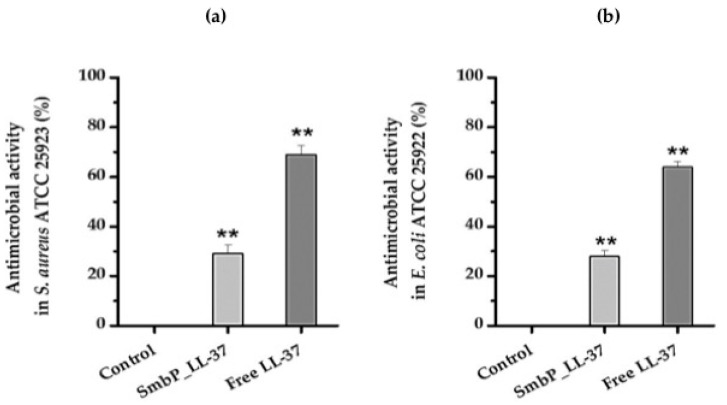
Antimicrobial activity of SmbP_LL-37 and free LL-37 using the CFU method. The antimicrobial activity was tested against (**a**) *Staphylococcus aureus* ATCC 25923 and (**b**) *Escherichia coli* ATCC 25922. A 50-mM Tris-HCl and 500-mM NaCl buffer was used as the negative control. The bars represent the averages of three independent experiments, and the error bars indicate the standard deviation of the means. Asterisks indicate statistically significant differences (**, *p* < 0.05 by Student’s *t*-test) compared to the control.

It is well-known that LL-37 has antimicrobial activity against many Gram-positive and Gram-negative bacteria. We selected *Staphylococcus aureus* and *Escherichia coli* to test the bactericidal activity of SmbP_LL-37 and free LL-37, as they are some of the most common pathogenic bacteria [25,26]. As shown in Figure 6, the complete SmbP_LL-37 protein showed antimicrobial activity against both bacterial species: *E. coli* (ATCC 25922) and *S. aureus* (ATCC 25923). SmbP_LL-37 reduced growth of *E. coli* to ~28% with practically the same effect against *S. aureus* (~29%), being the first time the antimicrobial activity of LL-37 is tested when it is still attached to a fusion protein. After tag cleavage, the antimicrobial activity of free LL-37 increased significantly, reaching ~64% for *E. coli* and ~69% for *S. aureus*. Kanamycin, used as the positive control, inhibits 100% of the population of both *S. aureus* and *E. coli* (data not shown) at the same concentration (50 μM).

Different fusion tags such as GST [19], Small Ubiquitin-like modifier (SUMO) [27], THX [16,17,24], CBM3 [18], and a THX-SUMO dual fusion system [28] have been used to produce LL-37 in *E. coli.* They all involve a more complex purification method than our technique, and a multistep purification process increases the time and costs and complicates the scaling-up process. Furthermore, to verify LL-37 antimicrobial activity, these fusion proteins had to be removed. Our method using SmbP therefore seems a more practical approach to screen the expression, purification, and activity of antimicrobial peptides.

Using GST as a fusion protein to express LL-37 has a relatively simple purification process, but the final LL-37 produced was 0.3 mg/L. The inability of GST to produce a high amount of soluble LL-37 combined with its size drastically reduces its final yield [19]. Using THX as a fusion tag, 4.3 mg/L of LL-37 can be produced [24]; however, this method required three purification steps and the use of Triton X-100 in the lysis buffer. The technique with CBM3 produced 1 mg/L, and the methodology also used a nonionic detergent to solubilize LL-37 [18]. In our work, we obtained 3.6 mg of SmbP_LL-37, from which 1.1 mg represented the free LL-37. This total amount is comparable with the previous protocols without the need for detergents or any other particular materials. The antimicrobial activity of LL-37 against a wide range of bacteria has been reported [14]; in this work, free LL-37 showed activity against *E. coli* and *S. aureus*, being slightly more effective against *S. aureus*. This result was expected as compared to previous studies where LL-37 was expressed recombinantly in *E. coli*. The full recombinant protein SmbP_LL-37 showed antimicrobial activity against both bacteria. Nevertheless, the inhibition percentage was considerably lower than the results obtained from the free peptide. We previously observed the same pattern with the Bin1b peptide, where SmbP_Bin1b showed 25% activity against *S. aureus* and around 60% for *E. coli.* As previously discussed for Bin1b, the small size of SmbP does not interfere with the ability of LL-37 to fully exert its antimicrobial activity [6].

We developed an expression system capable of producing free LL-37 in a one-step purification process and facilitated the screening of antimicrobial activity. However, if a pure LL-37 peptide is required, further purification using size exclusion chromatography, HPLC, or a combination of both can be used to obtain a purity over 95% [16,28].

## 3. Materials and Methods

DNA constructs. The pET30a vector was digested with NdeI and XhoI restriction enzymes. The DNA sequence that codifies for SmbP_LL-37 was codon-optimized for *E. coli* expression and synthesized by GenScript (Piscataway, NJ, USA). The sequence was digested with NdeI and XhoI, and ligated into pET-30a. The construct contains an Enterokinase cleavage site between the fusion protein SmbP and the LL-37 peptide for tag separation. *E. coli* strain DH5α was used for general cloning procedures and DNA maintenance. All constructs used in this work were fully characterized by sequencing.

Protein expression. The expression plasmid pET30a_SmbP_LL-37 was transformed into *E. coli* BL21(DE3)-competent cells. For small-scale expression experiments, a single colony was cultured at 37 °C and 200 rpm in 20 mL of LB broth supplemented with kanamycin (30 μg/mL). Once the OD_600_ reached 0.4–0.6, the protein expression was induced by adding 0.1-M IPTG to a final concentration of 0.1 mM. The culture was incubated for an additional 16 h at 37 °C. For 1-L expression, overnight cultures were used to inoculate LB–kanamycin in baffled flasks and incubated at 37 °C until the OD_600_ reached 0.4–0.6. IPTG was added to a final concentration of 0.1 mM, and again, the culture was incubated for 16 h at 37 °C.

Protein purification. Cells from the 1-L culture were harvested by centrifugation at 4 °C. The pellets were resuspended in lysis buffer (50-mM Tris-HCl and 500-mM NaCl, pH 8.0). The cells were disrupted using a bead beater and 0.1-mm glass beads for 8–10 cycles of 15 s, followed by intervals of 45 s for cooling. Cell debris from the lysate was removed by centrifugation at 24,500× *g* for 10 min at 4 °C. Recombinant SmbP_LL-37 was purified with IMAC using the ÄKTA Prime Plus FPLC system (GE Healthcare, Chicago, IL, USA). The supernatant was loaded into a HisTrap FF 1-mL column charged with Ni(II) and previously equilibrated with a lysis buffer; after sample loading, the column was washed with more lysis buffer. SmbP_LL-37 was eluted using an imidazole gradient (up to 200 mM) in 40 column volumes. The fractions were analyzed by SDS-PAGE or Tricine SDS-PAGE, the purity of SmbP_LL-37 being determined using ImageJ software (http://imagej.nih.gov/ij, accessed on 27 July 2021). Subsequently, the pooled fractions were dialyzed against the lysis buffer. After dialysis, 20 units of Enterokinase, his, and bovine (GenScript, Piscataway, NJ, USA) were used per milligram of SmbP_LL-37 for digestion in a 2-mL reaction volume for 16 h at room temperature. Protein concentrations were quantified using the Bradford reagent, with bovine serum albumin (BSA) as the standard.

Antimicrobial activity. The recombinant peptide was analyzed against *Staphylococcus aureus* (ATCC 25923) and *Escherichia coli* (ATCC 25922) using the colony-forming unit (CFU) assay method [29]. In brief, cell cultures in the log phase were diluted to a concentration of 10^6^ CFU per mL in sterile PBS buffer (10 mM, pH 7.4). Recombinant SmbP_LL-37, free LL-37, kanamycin (all at 50 μM), or the control buffer (50-mM Tris-HCl and 500 mM-NaCl, pH 8.0) was added to the bacterial suspension, and the cells were incubated at 37 °C for 3 h with shaking. After a 1000-fold dilution with a sterile phosphate buffer, 0.1-mL aliquots were spread on LB agar plates and incubated for 16 h to allow full colony development. After incubation, individual colonies of bacteria were manually counted. The ratio of colonies counted to the number of colonies on a control plate was calculated and averaged over three independent experiments. The antibacterial activity was described as the percentage of the population that was inhibited after the incubation.

## 4. Conclusions

In summary, we have reported the expression and purification of the antimicrobial peptide LL-37 with the SmbP fusion system. This strategy enabled rapid production with only one purification step for SmbP_LL-37, which showed activity against *S. aureus* and *E. coli.* After cutting with Enterokinase, the free LL-37 peptide increased its antimicrobial activity, as expected. Here, we offer a strategy that can be generally applied to more cationic peptides to quickly screen their antimicrobial properties against a wide variety of bacteria.
